# On the So-called “Wood Naphtha” of Dr. Hastings

**Published:** 1843-11-11

**Authors:** William Procter

**Affiliations:** Philadelphia


### ON THE SO-CALLED "WOOD NAPHTHA" OF DR. HASTINGS.

BY WILLIAM PROCTER, JR.

As notices of the medical characters of this substance have appeared in several of the journals, some observations on its chemical features will, perhaps, be acceptable to the readers of the Examiner. The term Naphtha, as applied to this substance, is incorrect, and properly belongs to the compound, $C^5 H^6$, used to preserve potassium; and some not aware of the difference have used the latter.

The editor of the London Pharmaceutical Journal has satisfactorily shown, in the July number of that periodical, that the naphtha of Dr. Hastings, is *acetone*, or *pyro-acetic spirit*, a substance formed by the decomposition of acetic acid when the acetate of a base having a strong affinity for carbonic acid is destructively distilled; the acid being resolved into acetone, which distils over, and carbonic acid which remains united with the base, thus: $C^4 H^3, O^3 = CO^2$ and $C^3 H^3 O$.

Acetone may be conveniently prepared, according to Graham, by distilling a mixture of two parts of acetate of lead, and one part of quick-lime, in an ordinary glazed earthen jar. The bottom and sides of the jar should be protected with fire clay, and the mouth closed accurately with a cork perforated with a large glass tube, so bent as to form a distillatory apparatus. The jar, thus protected, should be placed in the mouth of a small furnace, so that the lower portion of the vessel shall be heated to redness. A proper condensing apparatus should be attached to the glass tube,—an ordinary receiver, if kept cool with ice water, is quite sufficient.

In a recent trial of this formula, after the mixed powders were introduced into the jar, the temperature of the latter was observed to increase very considerably whilst vapour issued from the tube. This reaction appears to be due to the affinity of the anhydrous lime for the water of crystallization of the lead salt. The vapour was merely aqueous, containing no acetone. It is necessary to have that portion of the jar which is exposed to the heat well covered with some protecting material, as much of the product is lost in case the jar cracks; nearly one-half in one of my experiments with the process.

Crystallized acetate of lead should yield, theoretically, 15.3 per cent. of acetone by its decomposition, rather more than one-seventh; but, in practice, the product is rather less. It is probable that in England, where the article is largely manufactured, it is obtained either by decomposing acetate of lime in the establishments for making acetic acid from wood, or from the impure acid liquor, in which it occurs associated with pyroxylic spirit and other substances; and has probably obtained its title of "Wood Naphtha" from that circumstance.

Pure acetone is a colourless liquid, having a peculiar penetrating and empyreumatic odour. Its density in the liquid state is almost the same as that of alcohol: 0.7921. It is miscible in all proportions with water, alcohol, and ether; has a disagreeable taste, somewhat like peppermint; is highly inflammable, and boils at the temperature of 132° Fahr. (Graham.)

The "Wood Naphtha" employed in this city, which is of English origin, has a specific gravity of 0.820.

and burns with a blue flame; whilst acetone, according to Graham, burns with a white flame.

Supposing that the greater density of the commercial article was due to the presence of water, a portion of it was agitated with hydrate of potash, and another with fused chloride of calcium, and it was found to dissolve both substances; and when the solutions were inflamed, the residue, in both cases, gave evidence of water; the potash solution being partly liquid, and the chloride in the form of hydrated crystals.

As both of these salts are insoluble in acetone, it is highly probable that the commercial acetone in question contained either alcohol or pyroxylic spirit and water. It was less pungent and odourous than a specimen made for the occasion, and which was not wholly deprived of water.

*Philadelphia, Nov.* 1843.

